# Concomitant Chromosomal and Molecular Aberrations in Trisomy 8 Mosaicism and Associated Compound Phenotypes: Report of Three Cases and Review of Literature

**DOI:** 10.1155/crig/4494577

**Published:** 2026-01-29

**Authors:** Zakia Abdelhamed, Daniel Dykas, Autumn DiAdamo, Hongyan Chai, Deqiong Ma, Michele Spencer-Mazon, Yong-Hui Jiang, Jiadi Wen, Allen Bale, Peining Li, Hui Zhang

**Affiliations:** ^1^ Department of Genetics, School of Medicine, Yale University, New Haven, Connecticut, USA, yale.edu

**Keywords:** compound phenotype, concomitant aberration, pathogenic variant, Trisomy 8 mosaicism (T8M)

## Abstract

Trisomy 8 mosaicism (T8M) syndrome is a rare aneuploidy condition affecting 1/25,000–50,000 live births. Affected individuals have highly variable phenotypes from very mild dysmorphism to severe structural anomalies caused by chromosomal mosaicism and possibly undetected molecular aberrations. The utilization of chromosome microarray analysis (CMA) and exome sequencing (ES) in clinical laboratories enable the identification of genomic copy number imbalances and pathogenic gene variants. We presented one patient with a double aneuploid mosaic pattern of Monosomy X and Trisomy 8 for a compound phenotype of Turner syndrome (TS) and T8M syndrome, the second patient with T8M and a mosaic pathogenic variant in the *PTEN* gene detected by ES, and the third patient with typical phenotypic constellation of malformations with no other genetic aberrations detected by CMA and ES. Classification of mosaic findings was provided using a recommended six‐attribute scheme. Review of the literature summarized cases of T8M with concomitant molecular defects of a deletion at 22q11.2 and pathogenic variants in the *SALL1*, *RECQL4*, *NF1*, *CASK,* and *PAH* genes. These observations indicated that integrated cytogenetic and genomic analyses should be offered to patients with phenotypic abnormalities outside the spectrum of the T8M syndrome for comprehensive laboratory diagnosis and clinical management.

## 1. Introduction

In early 1970’s, a new syndrome with multiple congenital abnormalities was initially suspected in a small‐for‐gestational age male infant with a C‐group chromosome mosaicism and then formally reported as Trisomy 8 mosaicism (T8M) [1]. Full Trisomy 8 is embryonic lethal and accounts for 0.4% of chromosomal abnormalities in pregnancy loss [2, 3]; however, T8M is viable, albeit with highly variable presentations [4]. This T8M syndrome, also known as Warkany Syndrome 2, is very rare, and its incidence is estimated as 1:25,000–50,000 live born with a male‐to‐female ratio of 5:1 [5].

The development mechanism of T8M may be prezygotic meiotic nondisjunction with partial postzygotic loss of the extra Chromosome 8 or postzygotic mitotic nondisjunction. However, it appears that the most frequent mechanism of this condition is postzygotic mitotic nondisjunction error in a diploid conceptus, followed by random distribution of aneuploid cells between the different compartments of the developing embryo [6]. Disseminated (widely spread) or segmental mosaicism develop according to the timing at which the postzygotic error occurs. Very early postzygotic error led to disseminated mosaicism where multiple tissues are affected, while segmental mosaicism is due to late postzygotic event and usually affects a single‐tissue type. Disseminated mosaicism is the most common mechanism in autosomal aneuploidies including T8M. This would explain the mosaicism, survival, and good clinical prognosis of these patients compared to full trisomy patients. The affected subjects have a phenotype ranging from normal or near normal with mild facial dysmorphism to severe structural abnormalities. Craniofacial dysmorphisms, skeletal abnormalities, conductive hearing loss, ocular, renal and ureteral anomalies, brain anomalies, and congenital heart defects are commonly observed in T8M patients [7]. Additionally, patients with T8M are at increased risk of developing hypoplastic anemia, leukemia, and myelodysplastic syndrome, which may be due to altered stromal cell function leading to progenitor cell proliferation and expansion [8].

There is no known direct correlation between the proportion of Trisomy 8 cells and the severity of the phenotype. However, phenotypic variability could correlate with which tissues are most affected. The variable phenotype may also relate to modifying genetic factors or presence of another genetic aberration for a dual diagnosis. This study reports three patients of T8M brought for clinical genetics assessment of delayed development and abnormal facial features. Detailed cytogenetic analysis, chromosome microarray analysis (CMA), and exome sequencing (ES) were instrumental in providing a comprehensive genetic diagnosis in all three patients.

## 2. Case Presentation and Genetic Analyses

### 2.1. Diagnostic Workup

#### 2.1.1. Chromosome Analysis and Fluorescence In Situ Hybridization (FISH)

Karyotyping was performed on Giemsa–Trypsin–Wright’s (GTW) banded metaphases for blood lymphocytes or cultured skin fibroblast cells following laboratory’s standard protocols [9]. FISH tests were performed using probes for the FGFR1 gene at 8p12, the D8Z2 locus at Chromosome 8 centromere, the MYC gene at 8q24, and the centromeric region of Chromosome X (DXZ1) and a Yq12 locus (DYZ3) (Oxford Gene Technology Inc. UK). FISH signals from 100 to 200 interphase nuclei were scored using a fluorescence microscope [10].

#### 2.1.2. CMA

Genomic DNA was extracted from peripheral blood lymphocytes or cultured skin fibroblasts using the Gentra Puregene Kit (Qiagen, Valencia, CA). CMA was performed by array comparative genomic hybridization using Agilent SurePrint G3 Human CGH + SNP microarray (Agilent Technologies, Inc., Santa Clara, CA), as previously described [11]. The base designations were based on the February 2009 Assembly (GRCh37/hg19) of the UCSC Human Genome browser (https://genome.ucsc.edu/).

#### 2.1.3. ES

The extracted DNA was submitted to clinical ES on the Illumina platform as previously described [12]. GATK best practices are applied to identify genetic variants, and variants are annotated by ANNOVAR and a custom pipeline that includes allele frequencies, OMIM and ClinVAR citations, and numerous in silico attributes. The annotated data are analyzed for the presence of genetic variants among genes relevant to the patient’s phenotypes, and the variants are classified following ACMG/AMP guidelines [13].

CNV analysis from the ES data was performed as previously described [14]. Briefly, a proprietary program using read depth of exome captured regions normalized to read depth for each chromosome. The normalized sequence depths for a sample batch of six or greater samples were used to stratify target regions by variance, where those regions significantly vary from the batch means can be detected. Validation of this method and confirmation of the patients’ results were performed using CMA, chromosome, and FISH analysis, as described above.

#### 2.1.4. Classification of Mosaicism

Mosaicism is a condition that indicates an individual has at least two populations of cells with distinct genotypes that are derived from a single fertilized egg; clinically, a six‐attribute classification of genetic mosaicism has been proposed recently [15]. This classification scheme takes into consideration the affected cell type (A, A1 for somatic mosaicism), body pattern (B, B2 as disseminated), direction of change (C, C2 for pathogenic to benign and C3 for double mosaic conditions), developmental mechanism (D, D2 for segmental mosaicism with an early second hit, and D4 for disorders that manifest only as mosaics), etiology (E, E1 for large genomic changes, and E2 for small genetic changes), and fraction of the affected tissue (F, F1‐4 for mild, moderate, severe and very severe involvement, respectively).

Three patients presented in this study were seen after 2010 with referrals for genetic consult at Yale New Haven Hospital. All laboratory procedures were performed in certified cytogenetics and DNA diagnostic laboratories at the Department of Genetics in Yale School of Medicine. This project was categorized as a chart review retrospective study and deemed exempted from the Institutional Review Board (IRB) approval and granted waiver of consent based on the policy of the Yale University IRB.

### 2.2. Case Presentation and Analytic Results

#### 2.2.1. Patient 1

The first patient was presented as a female toddler with speech delay, frequent ear infection, and distinctive facial features including hypotelorism, broad philtrum, thick frenulum with wide spacing between teeth, and anterior override. She also had hockey stick palmar creases and convex nails as well as very dry skin, and an abnormal gluteal crease. This patient’s constellation of findings raised concern for conditions with midline abnormalities; however, brain MRI detected no midline cleft, and normal cardiac anatomy was detected by echocardiography. During the follow‐up visit, the patient was found to have improved speech with intensive speech therapy, but had hypotonia, joint hypermobility, and curved toes. The patient has primary ovarian insufficiency secondary to bilateral oophorectomy at age two. At the age of 3 year to 2 months, endocrine assessment revealed elevated FISH and decreased anti‐Mullerian factor. This patient was presented as a poster in the 2024 ACMG annual meeting [16].

Chromosome analysis and CMA analysis of the patient’s blood cells identified 45, X diagnostic for Turner syndrome (TS). Since the patient’s clinical picture did not perfectly match with typical TS phenotype, a skin biopsy and FISH testing on skin fibroblast cells were performed for evaluation of possible mosaicism. Indeed, a mosaic pattern was detected with Monosomy X (45, X) in 65% of the fibroblast cells and Trisomy 8 in male complement (47, XY, +8) in 35% of the fibroblast cells. Supplementary FISH analysis on peripheral blood sample confirmed a double aneuploid mosaic pattern of 45, X at 87.5% of leukocytes, and 47, XY, +8 at 12.5% of leukocytes. This result established the diagnosis of mosaic Monosomy X by a loss of Y chromosome and T8M in the XY cells. The unexpected detection of the Y‐chromosome material in fibroblast cells changed the patient management with prophylactic oophorectomy to avoid gonadoblastoma development. The presence of double mosaic conditions can explain the very unusual phenotype combination observed in this patient. According to the six‐attribute classification of mosaicism, the mosaicism in this patient could be designated as A1B2C3D2E1F2.

#### 2.2.2. Patient 2

The second patient was a male product of 36 weeks gestation and delivered via C‐section with a birth weight of 9 pounds. The pregnancy was complicated by preeclampsia. The postnatal course was complicated by NICU admission for 1 week due to respiratory distress, and the patient was otherwise well with no additional concerns. At his last evaluation at age 17, the patient had a weight of 65.2 kg (41%) and height of 172 cm (28%) and was referred to genetic consultation for evaluation of an arachnoid cyst, hammertoes, and learning disorder. Additionally, the patient was noted to have spinal asymmetry and lower limb length discrepancy. Interestingly, facial dysmorphism and deep palmar or plantar creases were not observed. The phenotypes reported were most consistent with multiple anomalies and intellectual disability.

Clinical ES detected two mosaic conditions in the patient’s blood peripheral lymphocytes: (1) a pathogenic variant in the *PTEN* gene, PTEN: NM_000314.8: c.209 + 1G > T (hg19:10:89685315) at 22% allelic fraction (Figure 1(a)) and (2) constitutional duplication of Chromosome 8, average copy number ratio is 1.29, indicating a mosaic pattern of this chromosomal abnormality (Figure 1(b)). CMA detected T8M (arr (8) × 3 [0.6]) in 60% of the blood lymphocytes. To determine if this double mosaic abnormality represented true constitutional mosaicism or due to an abnormal white blood cell clone, skin fibroblasts were analyzed by ES and cytogenetic testing. ES on DNA extracted from cultured fibroblasts detected both the *PTEN* variant and T8M, albeit at lower allelic fraction than what is detected in the blood lymphocytes. The *PTEN* c.209 + 1G > T detected at 11% allelic fraction (Figure 1(c)), and an average copy number ratio of 1.14 (T8M at 28%) were observed in the patient skin fibroblasts. Chromosome analysis detected mos 47, XY, +8 [12]/46, XY [8] with Trisomy 8 (Figure 1(d)) and a normal male complement (Figure 1(e)) in 60% and 40% of cultured fibroblasts, respectively. The patient also was referred to hematology evaluation, and complete blood count with differential was performed without evidence of malignancy. The presence of the second mosaic *PTEN* pathogenic variant would most definitely explain the atypical T8M presentation observed in this patient. The double mosaic pattern of this patient could be designated as A1B2C3D2E1&2F2.

Figure 1Exome sequencing identified mosaic variants in the blood lymphocytes and fibroblasts of Patient 2. (a) IGV image showing the mosaic *PTEN*: c.209 + 1G > T at the genomic location (hg19:10:89685315), detected at 23% VAF in lymphocytes (top raw), the middle and bottom are sequencing data from two control samples from the same sequencing run. (b) Line graph showing the allelic ratio of the exome CNV reads obtained from the patient (blue) and seven different control samples (CS‐1 through 7) (different colors). *Note*: Data from the patient clustered around 1.4, while that from the control samples seen around 1 as expected. (c) IGV image showing the mosaic *PTEN*: c.209 + 1G > T detected at 11% variant allele in fibroblasts (top raw), the bottom are sequencing data from another control sample from the same sequencing run. (d) Karyotype showing Trisomy 8 in fibroblast, and (e) Normal male karyotype in fibroblasts.

**Figure:**
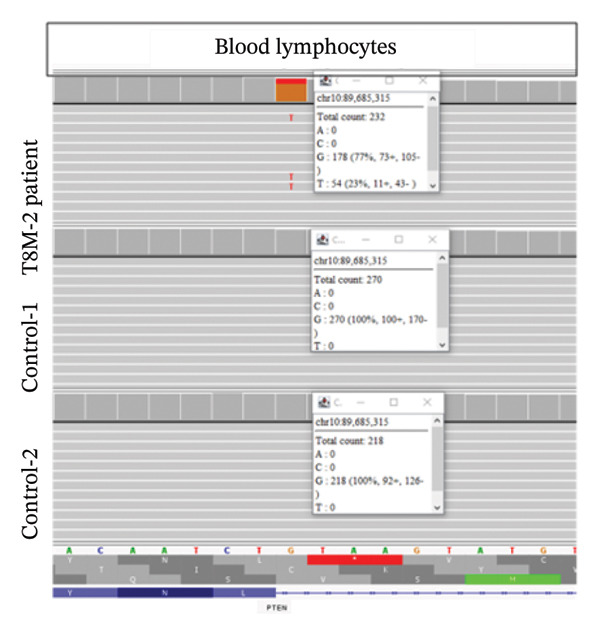
(a)

**Figure:**
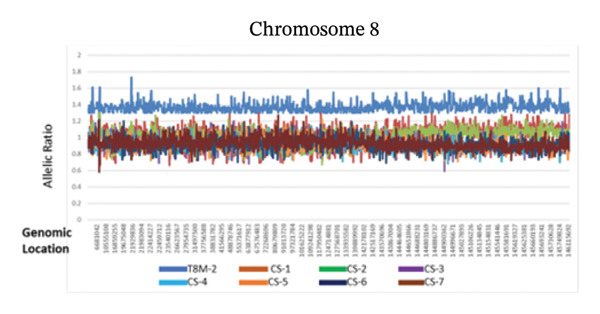
(b)

**Figure:**
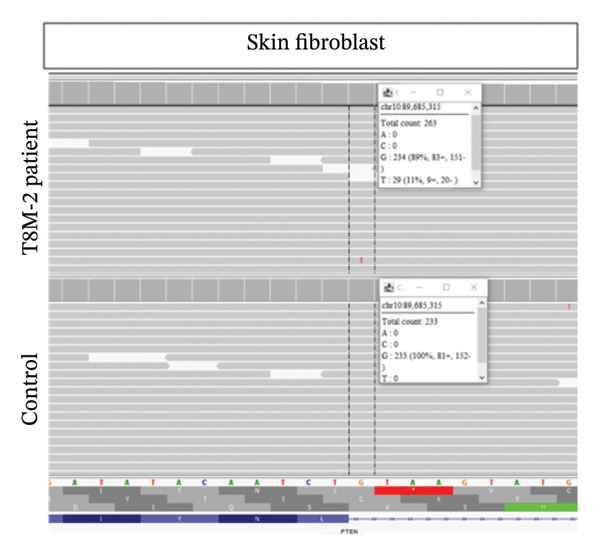
(c)

**Figure:**
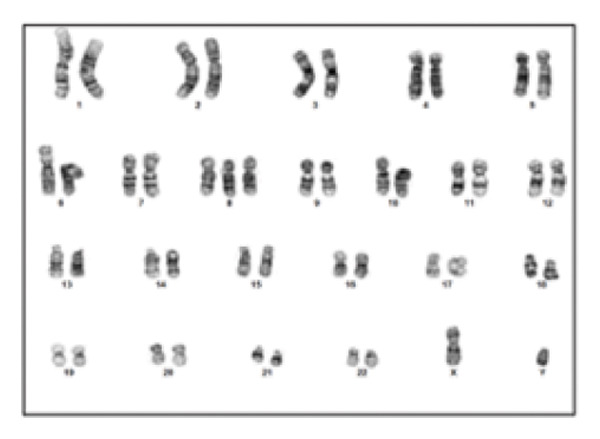
(d)

**Figure:**
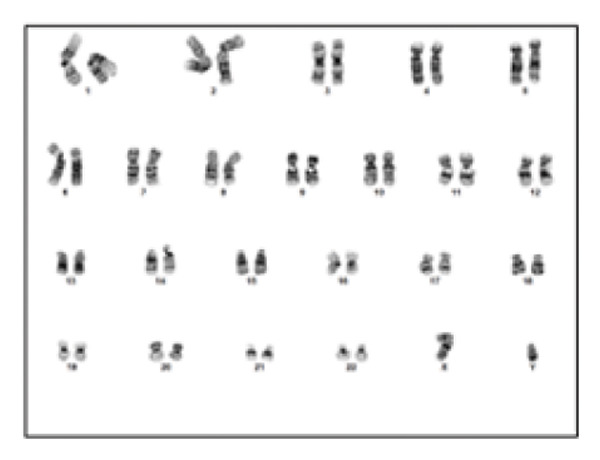
(e)

#### 2.2.3. Patient 3

The third patient was seen as a newborn male with dysmorphic features. The patient was a product of full‐term uneventful pregnancy to a 34‐year‐old female and spontaneous uncomplicated vaginal delivery. The birth weight was 3270 g. Noncontributory family history was noted except for TS (anatomic variant that poses risk for iliofemoral DVT) in the mother. On examination, the patient presented with facial dysmorphisms including prominent suborbital eye creases, ears flattened to side of the skull with a crease and prominent ear lobes, prominent nose with a flattened nasal tip, wide neck, short columella, short philtrum, and micrognathia but no cleft of palate. Other anomalies included bilateral deep palmar creases, rocker bottom feet deviated medially, and toes 2–4 bilaterally curved to the midline, hypoplastic toenails of the right 5th toe, and absence of toenail on the left.

CMA revealed an XY male with a mosaic pattern of a 146.294 Mb duplication at 8p23.3q24.3 (chr8:161,472–146,294,098, L2R:0.287) for the entire Chromosome 8. Based on the log2 ratio (L2R), it was estimated that T8M is about 44% of the cells. The FISH test using probes for the FGFR1 gene and the D8Z2 locus detected three fusion signals for the FGFR1 probe and three signals for the D8Z2 probe in 20% of patient’s blood cells. Karyotyping confirmed the T8M detected in more than 50% of the cells analyzed. As expected, clinical ES CNV analysis detected Trisomy 8 with an average mean dosage of all exons across the whole Chromosome 8 equivalent to 1.18 (∼36% of cells with T8M). However, no other clinically relevant single‐nucleotide variants were detected in this patient’s blood DNA. The classification of this T8M condition was A1B2C1D4E1F2.

During regular follow‐up visits up to age of 3 years, the patient developed a long face with dolichocephaly, epicanthal folds, high‐arched eyebrows, large low set ears with thick lobes bilaterally, bulbous nose with wide nasal bridge and root, upturned nostrils, large mouth with widely spaced teeth, and frontal bossing with long forehead. The pediatric echocardiogram was essentially normal apart from a small patent foramen ovale with left to right shunt. Skeletal survey showed no kyphosis, scoliosis, or patellar abnormalities. However, bilateral coxa valga and pubic symphysis diastasis were noted. Neuropsychiatric assessment indicated that the patient had global developmental delay including language delay and toe walking. The patient was also reported to have disrupted sleep maintenance.

### 2.3. Review of Literature for T8M Cases With Concomitant Aberrations

Review of the literature noted several cases of T8M with concomitant molecular aberrations. A newborn presenting complex congenital cardiac defects and succumbed to cardiac arrest at 2 weeks of age was detected with T8M and a deletion at 22q11.2 for DiGeorge syndrome [17]. A 14‐year‐old girl was detected with T8M syndrome and Townes–Brocks syndrome due to a novel pathogenic variant in the *SALL1* gene [18]. A 6‐month‐old boy presenting dysmorphic features and café‐au‐lait spots was detected with T8M and a pathogenic variant in the *NF1* gene for neurofibromatosis Type 1 [19]. Patients with Rothmund–Thomson syndrome due to pathogenic variants in the *RECQL4* gene were noted with concomitant T8M [20], and two adult brothers with these defects showed compound phenotypes [21]. A 5‐year‐old girl presenting developmental delay, autistic behavior, hyperplasia of the cerebellum and brainstem, and café‐au‐lait spots was detected with T8M, a maternally inherited pathogenic variant in the *NF1* gene, and a *de novo* pathogenic variant in the *CASK* gene [22]. Recently, a 22‐year‐old female with intellectual disability and slurred speech was detected with T8M and heterozygous pathogenic variants in the *PAH* gene for phenylketonuria [23]. The cytogenetic and molecular genetic aberrations and compound phenotypes in these cases and our Patient 2 are summarized in Table 1. More than 120 cases of T8M have been reported [24], thus an estimation of more than 6% of T8M patients could have concomitant molecular aberrations.

**Table 1:** Cases of T8M with concomitant molecular aberrations.

Case no.	Gender and age	Chromosome mosaicism	Pathogenic CNV and gene variants	Compound phenotypes	References
1	F, 2 wk	mos 47, XX, +8 [23%]/46, XX [77%]	Del (22) (q11.2)	T8M syndrome/DiGeorge syndrome	[17]
2	F, 14 y	mos 47, XX, +8 [8]/46, XX [12]	SALL1: c.2779C > T	T8M/Townes–Brocks syndrome	[18]
3	M, 6 m	mos 47, XY, +8 [90%]/46, XY [10%]	NF1: c.1019_1020delCT	T8M syndrome/NF1	[19]
4, 5	M, 34 y and 39 y brothers	T8M in 15% and 13% of lymphocytes	RECQL4: c.1048_1049delAG; c.1391‐1G > A	Rothmund–Thomson syndrome/mild T8M	[20, 21]
6	F, 5 y	mos 47, XX, +8 [3]/46, XX [47]	NF1: c.6060dupA CASK: c.2041C > T	T8M syndrome/NF1/CASK	[22]
7	F, 22 y	mos 47, XX, +8 [10]/46, XX [104]	PAH: c.611A > G; c.1256A > G	Mild T8M syndrome/phenylketonuria	[23]
8	M, 17 y	mos 47, XY, +8 [12]/46, XY [8]	PTEN: c209+1G > T	T8M syndrome/PTEN	This study (Patient 2)

## 3. Discussion

A spectrum of variable phenotypes has been reported in the T8M syndrome [6, 7]. Craniofacial dysmorphisms include prominent forehead, deep‐set eyes, strabismus, broad nasal bridge, upturned nares, long upper lip, thick and everted lower lip, high‐arched or cleft palate, micrognathia, corneal opacities, and large dysplastic ears with prominent antihelices. Skeletal abnormalities include slender trunk, short or webbed neck, abnormal scapula and sternum, widely spaced nipples, hemivertebrae, spina bifida, kyphoscoliosis, hip dysplasia, multiple joint contractures, camptodactyly of the second through the fifth fingers and toes, dysplastic nails, limited elbow supination, and absent or dysplastic patella. Conductive hearing loss, renal and ureteral anomalies, cryptorchidism, uterus didelphys, jejunal duplication, agenesis of the corpus callosum, and congenital heart defects are commonly observed in T8M patients. It has been observed that T8M could affect chromatin processing and compartment as well as epigenetic modification on Chromosome 8 with altered gene dosage effect for clinical features [25].

Patients of T8M with atypical and additional phenotypes could raise concern of undetermined genetic aberrations. Our first patient with T8M in male cells is like previously reported cases with double‐aneuploid mosaicism of T8M and Monosomy X [26, 27]. In fact, this dual chromosomal mosaicism seems more common than expected; a study showed the occurrence of T8M in 4% of 152 TS patients [26]. Recently, an 11‐year‐old child showing prominent forehead, hypertelorism, deep set eyes, wide and anteverted nose, dysplastic ears, thick lips, short and wide neck, ans accessory nipple; pigmentary anomalies follow that Blaschko’s lines was detected with chimerism of chi 47, XY, +8/46, XX in cells from blood, skin, and testis [27]. Double mosaicism for Trisomy 8 and Trisomy 21 was reported in a male child with Warkany–Down compound phenotypes [28]. A patient with T8M detected in skin fibroblasts but not in peripheral blood was reported, suggesting likely a postzygotic mitotic error and selective growth advantage for intertissue difference, as seen in our Patient 1 [29, 30]. Overall, these cases may suggested possible chromosomal interference and likely explained the known male preponderance of the T8M condition and a natural selection against Trisomy 8 in female conceptuses or cells [30]. Additionally, the level of mosaicism and its correlation to specific phenotypes should be taken into consideration in future studies.

Our second patient’s phenotype description was not pointing to a specific genetic condition, and therefore an integrated diagnostic approach was applied. To our knowledge, this is the first reported case of a double mosaicism of a pathogenic *PTEN* variant and the T8M. *PTEN* hamartoma and Cowden syndromes due to *PTEN* mosaic pathogenic variants is a classic example of Type 2 segmental mosaicism of autosomal dominant traits [31, 32]. The constitutional nature of this double mosaic condition was confirmed by testing the patient’s skin fibroblasts. Learning disability is common in both *PTEN* and T8M patients. However, lower limb length discrepancy is a feature of segmental overgrowth syndromes such as Proteus syndrome and Proteus‐like syndrome caused by mosaic variants in the *AKT1* and *PTEN* genes, respectively [33]. The mosaic *PTEN* variant could explain the lower limb length discrepancy. The arachnoid cyst may also relate to the *PTEN* variants and has been reported in *PTEN* hamartoma syndrome patients [34]. The *PTEN*/*PI3K*/*AKT* pathway plays crucial roles in regulating cellular proliferation, metabolism, and survival; the phenotype seen in this patient could be explained by the concomitant mosaic variant in the *PTEN* gene. Overall, all reported cases of T8M with other concurrent variations involve genes presenting dosage‐sensitive effect or regulating development for compound phenotypes [17–23].

Our patients and cases reported in the literature demonstrated the importance of integrated cytogenetic and molecular analyses for concomitant chromosome mosaicism and pathogenic gene variants [35]. Routine chromosome analysis can detect T8M in cultured cells but could miss low percentage mosaicism. Locus‐specific FISH can be applied onto directly prepared cells to determine levels of mosaicism from various tissues. CMA and ES can detect pathogenic copy number and gene variants and estimate the allelic fraction in DNA content. The six‐attribute classification of genetic mosaicism should be used in the interpretation of mosaic findings [15]. Follow‐up cytogenetic and molecular analyses to track the change of mosaicism in the patients and to rule out familial carriers should be recommended. Detailed clinical evaluation to dissect the compound phenotypes and to provide actionable treatment and proper management should be considered.

In conclusion, patients with T8M may have intrinsic molecular aberrations leading to compound phenotypes. Cell‐based karyotyping and FISH could provide accurate evaluation of chromosomal mosaicism. DNA‐based CMA and ES could uncover pathogenic copy number variants and gene variants that may explain phenotypic variability. Knowledge of concomitant aberrations and their correlations to compound phenotypes are invaluable information that usually led to modification of treatment and/or surveillance.

## Author Contributions

Zakia Abdelhamed collected T8M cases and drafted the original manuscript; Zakia Abdelhamed, Daniel Dykas, Deqiong Ma, Allen Bale, and Hui Zhang performed ES and interpreted results; Autumn DiAdamo, Hongyan Chai. Jiadi Wen, and Peining Li contributed to chromosome, CMA, and FISH analyses and interpretation; Michele Spencer‐Mazon and Yong‐Hui Jiang performed clinical evaluation. Peining Li and Hui Zhang revised and edited the manuscript.

## Funding

No funding was applied to this case study.

## Disclosure

All authors reviewed and approved the manuscript.

## Ethics Statement

No written consent has been obtained from the patients as there are no patient identifiable data included in this case report. This project was categorized as a chart review retrospective study and deemed exempted from the Institutional Review Board (IRB) approval and granted waiver of consent based on the policy of the Yale University IRB.

## Conflicts of Interest

The authors declare no conflicts of interest.

## Data Availability

The data that support the findings of this study are available from the corresponding authors upon reasonable request.
